# The KUYUY Accelerograph and SIPA System: Towards Low-Cost, Real-Time Intelligent Seismic Monitoring in Peru

**DOI:** 10.3390/s26010254

**Published:** 2025-12-31

**Authors:** Carmen Ortiz, Jorge Alva, Roberto Raucana, Michael Chipana, José Oliden, Nelly Huarcaya, Grover Riveros, José Valverde

**Affiliations:** 1 Research Center for Digital Transformation in Engineering, Universidad Nacional de Ingeniería, Lima 15333, Peru; jalvah@uni.edu.pe (J.A.); rraucanas@uni.pe (R.R.); mchipanai@uni.pe (M.C.); joliden@uni.edu.pe (J.O.); nhuarcaya@uni.edu.pe (N.H.); griveross@uni.pe (G.R.); jvalverdea@uni.edu.pe (J.V.); 2 Faculty of Engineering, Universidad Peruana de Ciencias Aplicadas, Lima 15023, Peru

**Keywords:** accelerograph, MEMS sensors, SIPA, seismic monitoring

## Abstract

Accelerographs are essential instruments for quantifying strong ground motion, serving as the foundation of modern earthquake engineering. In Peru, the first accelerographic station was installed in Lima in 1944; since then, various institutions have promoted the expansion of the national network. However, this network’s spatial coverage and instrumentation remain insufficient to properly characterize strong motion and support seismic risk reduction policies. In this context, the KUYUY accelerograph is presented as a low-cost, low-noise device equipped with real-time telemetry and high-performance MEMS sensors. Its interoperability with the Intelligent Automatic Processing System (SIPA) enables real-time monitoring and automated signal analysis for seismic microzonation studies and rapid damage assessment, contributing to seismic risk reduction in Peru. The validation process included static gravity calibration, field comparison with a reference accelerograph, and an initial deployment in Lima and Yurimaguas. The results demonstrate the proposed accelerograph’s linear response, temporal stability, and amplitude consistency with respect to high-end instruments, with differences below 5–10%.

## 1. Introduction

### 1.1. Overview of International Accelerographic Networks

Accelerographs have been essential tools for studying strong ground motion since their appearance in the twentieth century. These devices initially operated using analog and mechanical systems, recording seismic accelerations on paper rolls or tapes requiring detailed visual interpretation. Hudson [1] has described in detail how these readings were performed. These early instruments made it possible, for the first time, to quantify seismic motion over time, thus laying the foundation for modern earthquake engineering.

Over time, seismic instrumentation has evolved significantly. Trifunac and Todorovska [2] have provided a technical review of accelerograph evolution, from the first mechanical systems to modern high-resolution, low-noise digital devices. They emphasized progressive improvements in data processing and in the spatial resolution of instrumented networks. A commemorative paper celebrating 75 years of strong-motion observations explored how the quality and quantity of records have directly influenced the development of earthquake engineering, and how these advances have been reflected in international technical standards [3].

In parallel with instrument evolution, accelerographic networks have also modernized. Italy provides a representative case: Michelini et al. [4] have described how the Istituto Nazionale di Geofisica e Vulcanologia (INGV) established a nationwide network of broadband and strong-motion stations integrated with automatic alert and analysis systems, providing critical information in real time. Another complementary study [5] analyzed network performance between 1985 and 2002, showing reduced source-to-station distances, improved event localization, and optimized site-selection criteria through geological and geotechnical investigations.

Regulatory frameworks have evolved alongside these developments. Çelebi et al. [6] have proposed a seismic instrumentation model for federal buildings in the United States, emphasizing the need for multiple sensors distributed along the height and base of structures. Their work considers soil–structure interactions and recommends configurations for accurately evaluating structural performance. Similarly, FEMA P-1050-1 [7] compiles scientific advances and translates them into design recommendations for new buildings, including the mandatory use of accelerographic records for response analysis.

Regulatory progress and network enhancement have been complemented by recent research focused on developing accessible technologies aimed at extending seismic instrumentation to contexts with limited budgets. In this scenario, low-cost accelerographs have emerged as a promising solution. These instruments can deliver performance comparable to commercial systems, but at a fraction of the cost and with greater ease of implementation. Evans et al. [8] have compared several low-cost MEMS sensors with traditional devices such as the Kinemetrics SMA-1. They found that some modern devices even outperform historical models in spectral resolution and linear response, enabling their use in community-based networks such as QCN and CSN.

A large-scale application example is the Community Seismic Network (CSN) deployed in Los Angeles. Clayton et al. [9] have described how a dense array of IoT-connected MEMS accelerographs can generate detailed strong-motion data within seconds. This network complements official USGS systems and enhances tools such as ShakeMap and ShakeCast.

Integrated device development has made it possible to combine MEMS sensors with modern communication protocols. Di Nuzzo et al. [10] have presented a structural monitoring system based on MEMS and NB-IoT connectivity, achieving precision comparable to that of commercial piezoelectric sensors. This system operates at a fraction of the cost and maintains more than ten years of autonomy using solar power and optimized batteries.

Similarly, De Alteriis et al. [11] have proposed a solution for large-scale positioning and structural monitoring using RTK-configured GPS modules and MEMS sensors. Their system employs Kalman filters to estimate structural displacements in real time with high accuracy. This approach is suitable for implementation in critical infrastructure such as bridges and tunnels, where installing conventional equipment can be challenging or costly.

A key development in this field is the QuakeFlow system proposed by Zhu et al. [12], which demonstrates how deep learning models can be integrated with MEMS sensors to identify seismic events not recorded in conventional catalogs. Complementing these advances, Papanikolaou et al. [13] have designed a dense network of low-cost triaxial MEMS accelerographs for urban seismic hazard assessment. The system is autonomous, easily configurable, and capable of local post-processing.

From a structural monitoring perspective, Xie et al. [14] and Crognale et al. [15] have proposed architectures that integrate MEMS sensors with synchronization algorithms and high-resolution data acquisition protocols. The former combines GNSS and MEMS sensors in bridges, while the latter developed a robust system that was validated on historical structures, showcasing the potential of these sensors for continuous monitoring.

In terms of instrument design, Vlachos et al. [16] have presented a low-cost seismograph capable of detecting local seismicity with high resolution, whereas Hasani et al. [17] have developed a robust wireless system and validated it through Operational Modal Analysis (OMA) on arch bridges, demonstrating its synchronization, energy efficiency, and reliability in real environments.

Furthermore, Scafidi et al. [18] and Komarizadehasl et al. [19,20] demonstrated the scalability of MEMS-based seismic networks in real-world environments. The former deployed a dense ADXL355-based accelerometric network in populated areas of Trentino (NE Italy) and integrated its recordings with permanent stations to automatically generate quasi-real-time shaking and exposure maps for civil protection. The latter applied low-cost MEMS accelerometers to urban bridges, showing that modal analysis and digital model validation are feasible using synchronized Arduino-based sensors.

Moreover, Paziewski et al. [21] and Saravanan et al. [22] have addressed GNSS integration and IoT node implementation, respectively. The former achieved submillimeter accuracy in dynamic displacement measurements, while the latter developed a DIY IoT-based network for laboratory-scale damage detection, showing that IoT approaches are viable, cost-effective, and scalable for real-time structural monitoring.

Although these studies confirm the maturity of MEMS-, IoT-, and GNSS-based systems, modern international reference strong-motion networks still rely primarily on force-balance accelerometers due to their high dynamic range, low noise levels, long-period stability, and flat broadband response. National networks in countries such as Italy, Japan, the United States, and Taiwan operate dense arrays equipped with State-of-the-Art digital instrumentation. The Italian RAN network, for example, uses Kinemetrics Episensor and FBA-23 accelerometers, which are considered the current standard. Costa et al. and Gorini et al. [23,24] extensively document the performance and quality of this type of instrumentation.

In parallel, Bragato et al. [25] presented the implementation of a dense MEMS-based accelerometer network in the Veneto region (Italy), designed to record low-magnitude seismic motions and to support rapid seismic impact assessment using near–real-time data. The network covers 312 locations and provides high-quality, low-cost measurements integrated with efficient data acquisition and management systems.

These technological advances establish the foundation for the design and validation of new in-house accelerographs, such as the three-axis accelerometer developed by our team, which directly addresses the challenges of cost, autonomy, and connectivity identified in the recent literature.

### 1.2. National Background of Accelerographic Instrumentation in Peru

Peru’s first permanent accelerographic station was installed in 1944 at Parque de la Reserva (Lima), using a strong-motion accelerograph provided by the U.S. Coast and Geodetic Survey in cooperation with the Geophysical Institute of Peru (IGP). Between 1946 and 1972, this station recorded 22 earthquakes, including the 31 May 1970 event (Mw 7.9), for which three-component accelerograms (two horizontal and one vertical) were preserved. The horizontal peak ground accelerations ranged from 0.10 to 0.11 g, making it the most iconic record in the station’s modern history [26,27,28].

After the 1970 earthquake, the IGP installed a Kinemetrics SMA-1 accelerograph in Lima in 1972. It recorded the 1974 earthquakes at various sites (Zárate, Casa del Dr. Huaco, and La Molina), marking the beginning of gradual instrumentation expansion in the capital [29].

Regarding representative earthquakes: (i) The 2001 Arequipa earthquake (Mw 8.4) was recorded in Moquegua using a RION SM-10B accelerograph, with a horizontal PGA of about 0.30 g; including northern Chile, seven strong-motion stations recorded the event [30]. (ii) The 2007 Pisco earthquake (Mw 7.9) triggered 18 Peruvian accelerographic stations, mostly located in Lima and two in Ica, reflecting the transition towards digital networks [31].

Since 2014, several institutions—including the College of Engineers of Peru, SENCICO, and the Allpanchis Research and Development Center (CIDALL)—have implemented an automated, real-time accelerographic network comprising over 80 stations. Within this framework, CIDALL researchers have developed an automatic monitoring system called SIPA. During the May 26, 2019, Lagunas earthquake (Mw 8.0), the network recorded data from 44 stations, reporting a maximum PGA of 95.84 cm/s^2^ at the UNTRM–Chachapoyas station [32].

Meanwhile, the IGP has progressively increased the number of accelerographs and initiated the automation of its network. Nevertheless, the total number of stations currently operating in the country remains insufficient for adequate characterization of strong ground motion.

In this context, the previous developments highlight important contributions from national institutions and universities; however, gaps remain in the spatial coverage of accelerographs in Peru and in the instrumentation of buildings. The earthquakes of 2001, 2007, and 2019 demonstrated the need for dense networks and timely telemetry. Although commercial force balance accelerographs represent the international standard due to their wide dynamic range and superior long-period stability, their high acquisition, operation, and maintenance costs limit large-scale deployment in the country.

Under these constraints, the KUYUY accelerograph, a low-cost, low-noise device operating with real-time telemetry, constitutes a viable, robust, and technically consistent solution for increasing the density of national seismic instrumentation. Its interoperability with automatic processing pipelines through the SIPA system facilitates microzonation studies and rapid damage assessment, contributing to seismic risk reduction. The maturity of high-performance MEMS sensors and international experience with dense arrays support this approach, providing a solid foundation for scalable and sustainable deployment in Peru [9,10,14].

## 2. Methodology

The methodological approach adopted in this study reflects the progressive and integrated development of an intelligent seismic monitoring system that combines automated data processing with dedicated low-cost instrumentation. The work initially focused on the development and implementation of the Intelligent Accelerographic Processing System (SIPA), which was designed to operate using data from commercial strong motion accelerographs. Since 2016, SIPA has been tested with real accelerometric records, enabling the implementation and validation of automated techniques for signal detection, noise discrimination, and extraction of engineering-relevant seismic parameters. Its operational robustness was notably demonstrated through its application to significant seismic events, including the 26 May 2019, Lagunas earthquake.

The experience gained from these applications highlighted the need for denser accelerometric coverage, particularly in regions such as Peru, where the limited number of permanent strong motion stations and the high cost of commercial accelerographs restrict large-scale deployment. This motivated the subsequent design and development of a dedicated low-cost accelerograph capable of operating seamlessly with SIPA.

Based on these requirements, the KUYUY accelerograph was designed and developed. KUYUY, named after the Peruvian Quechua word meaning “to shake”, is an accelerographic device equipped with high-performance MEMS sensors and real-time telemetry. Its development involved hardware design, system integration, and laboratory and field testing, with emphasis on reliability and full compatibility with the SIPA processing environment.

Within this framework, the complete seismic monitoring system operates through an integrated sequence. The KUYUY accelerograph provides continuous ground motion acquisition. The recorded data are transmitted in real time to a central server through a dedicated communication architecture. SIPA then performs intelligent automated processing, including signal detection, noise filtering, feature extraction, and real-time computation of parameters relevant for seismic and engineering applications. The interaction between KUYUY and SIPA results in a unified, low-cost, real-time, intelligent seismic monitoring system.

The following subsections describe in detail the design of the KUYUY device, the data acquisition and transmission architecture, and the Intelligent Accelerographic Processing System SIPA.

### 2.1. Design of the KUYUY Device

The KUYUY accelerograph is a compact three-axis accelerometer designed for urban and rural deployments while requiring minimal technical intervention, emphasizing mechanical robustness, electronic efficiency, and autonomy. It includes an anodized aluminum enclosure with IP68 protection (148 mm × 108 mm × 75 mm), leveling feet, and visible bubble levels for accurate alignment of the X, Y, and Z axes.

**Figure 1:**
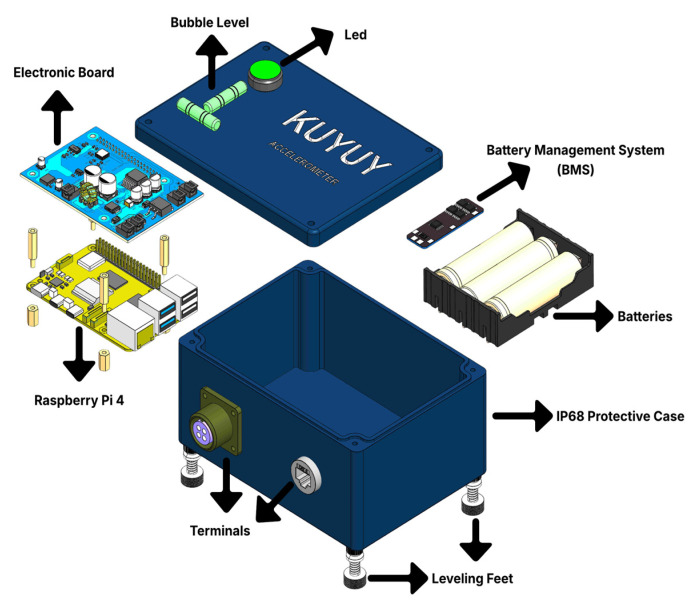
Exploded view of the KUYUY accelerograph showing internal components and installation elements.

Inside, it integrates a high-resolution MEMS accelerometer (20-bit; ±2 g/±4 g/±8 g) coupled to an embedded unit that manages data acquisition, storage, compression, and transmission. Ethernet and Wi-Fi connectivity enable remote management. The power architecture combines a 12 V input with battery backup managed by a Battery Management System (BMS). LED indicators and a reset button allow for manual supervision and recovery. These components are illustrated in Figure 1, which shows an exploded view of the modular design for simplified assembly, maintenance, and field deployment.

#### 2.1.1. Mechanical Design

**Figure 2:**
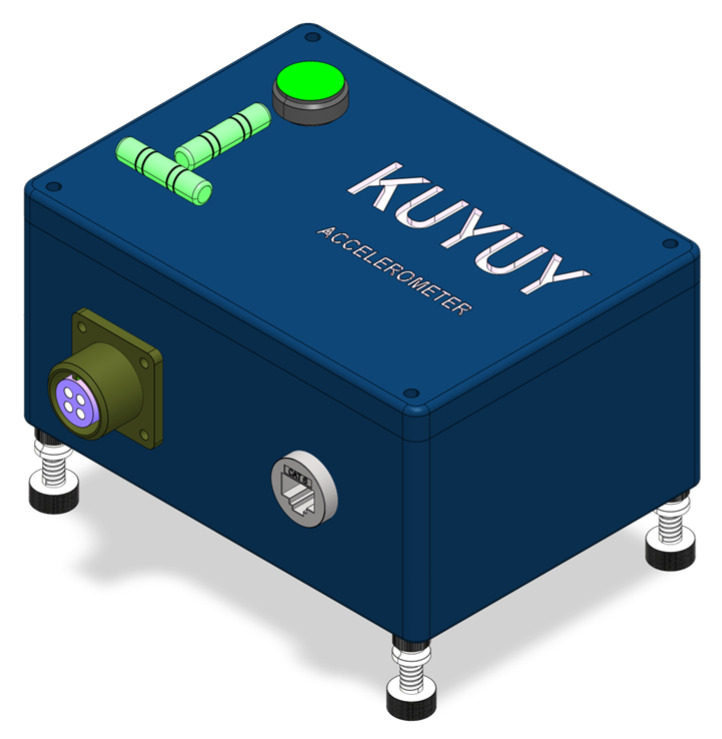
Assembled KUYUY accelerograph showing the enclosure, leveling feet, and signal/power connectors.

The anodized aluminum enclosure ensures IP68 protection, shielding the device from dust, water, and vibration during field operation. The design includes sealed entries for industrial connectors and anchor bolts for surface mounting, as shown in Figure 2.

Leveling and orientation are achieved through four threaded feet located at the corners of the base, allowing for precise horizontal alignment. Two bubble levels visible from the exterior facilitate orientation verification during installation, ensuring orthogonality between sensor axes and the monitored system.

The anchoring system at the base uses a quick-release sliding-slot mechanism. The enclosure slides over the head of a pre-installed bolt and, as it advances along the guide, the head is locked in a narrow section that prevents vertical detachment. This mechanism provides a stable yet reversible fixation, enabling fine leveling by adjusting the feet until the bubbles are centered.

#### 2.1.2. Electronic Design

The KUYUY architecture integrates an embedded processing unit (Raspberry Pi 4 Model B, Raspberry Pi Ltd.) and a triaxial MEMS sensor (ADXL355, Analog Devices, Inc.) connected via SPI on a double-layer PCB that includes signal conditioning and power regulation. The configuration is compact, modular, and reliable for field operation.

The processing unit, which is based on a Raspberry Pi 4 Model B (Broadcom BCM2711, quad-core Cortex-A72 at 1.5 GHz, 2 GB RAM), runs a Linux-based embed-ded operating system. The Raspberry Pi 4 is developed by Raspberry Pi Ltd. and is manufactured primarily at the Sony UK Technology Centre in Pencoed, Wales, United Kingdom. It coordinates device functions and manages Ethernet/Wi-Fi communications, as shown in Figure 1, where the embedded unit is presented as the main pro-cessor of the accelerograph.

The triaxial ADXL355 sensor provides an effective resolution of 20 bits, low noise (down to 25 μg/√Hz), thermal stability within ±0.5 mg/°C, and selectable ranges of ±2 g, ±4 g, and ±8 g. The sensor is designed and manufactured by Analog Devices, with MEMS fabrication and packaging facilities located in the United States and Ireland (notably Limerick, Ireland). It includes internal digital filters and shows good long-term stability, making it suitable for continuous structural monitoring. Communication with the embedded unit is carried out through SPI. The sampling frequency is configurable up to 4 kHz; for this study, a nominal rate of 200 sps was used for continuous recording.

The double-layer acquisition and conditioning PCB mounts the ADXL355 sensor with low-noise LDO regulators and the necessary power and signal components. MOLEX connectors and protection filters ensure signal integrity against external interference. Its compact, modular design facilitates integration with the Raspberry Pi, as shown in the system overview of Figure 1.

The KUYUY power system allows operation from an external 12 V DC source or an internal backup pack of three 18650 batteries in series (3S). The BMS manages charging, discharging, and automatic switching, prioritizing the external source when available. Low-dropout (LDO) regulators and operating system optimizations reduce power consumption by disabling unnecessary processes, minimizing energy demand from both the sensor and the embedded unit.

The system can operate autonomously for up to eight hours without external power. With further optimization, autonomy exceeds ten hours under test conditions. A set of LEDs indicates power and activity status, and a reset button enables manual recovery from critical failures.

### 2.2. Data Acquisition and Transmission to the Central Server

The embedded system continuously acquires accelerometric data at a rate of 200 samples per second (sps). Acquisition is synchronized with UTC, ensuring precise temporal accuracy. A new binary file (.bin) is generated every five minutes, which contains the three orthogonal components (X, Y, Z) with their respective timestamps. This segmentation scheme facilitates data organization and its later integration into large-scale processing platforms.

Upon completion, the files are compressed into .bin.gz format and transferred to remote servers via FTP. In case of connection loss, the files remain stored locally until communication is restored, ensuring data integrity and continuity. The system also performs automatic cleanup, deleting files older than six months to preserve storage capacity while retaining recent records for local access.

Operational robustness is maintained using systemd, which monitors critical services and restarts them automatically after power failures or operating system interruptions. This approach guarantees the continuous and resilient operation of acquisition, compression, and transmission processes.

### 2.3. Intelligent Accelerographic Processing System (SIPA)

The Intelligent Accelerographic Processing System (SIPA) is a dedicated computational framework designed to automate the processing and analysis of accelerometric records acquired with multiple accelerograph models, including KUYUY instruments. Its primary objective is to transform raw ground-motion recordings into engineering-relevant seismic parameters through a structured sequence of classification, correction, and spectral analysis procedures.

SIPA comprises three major functional components:

- Automatic Seismic–Non-Seismic Discrimination: A deep learning classifier distinguishes ground motions produced by earthquake activity from those generated by non-seismic sources, such as traffic, industrial machinery, wind, or electronic interference.
- Signal Correction and Conditioning: The system performs baseline removal procedures, applies band-pass filtering, and suppresses instrumental noise to ensure the physical consistency of the processed acceleration records.
- Computation of Seismic Engineering Parameters: From the corrected three-component acceleration signals, SIPA derives peak ground acceleration (PGA), peak ground velocity (PGV), Arias intensity, maximum displacement, and elastic response spectra, which are relevant for structural and seismological analyses.

Through these components, SIPA systematically converts raw accelerometric inputs into high-quality, analysis-ready ground-motion datasets suitable for engineering and hazard assessment applications.

#### 2.3.1. Data Collection

For model training purposes, accelerometric records collected between 2016 and 2018 were assembled, comprising more than 12,000 three-component ground-motion signals. Approximately 2200 of these were associated with confirmed seismic events, while the remaining records corresponded to non-seismic vibrations. Each record included the three orthogonal components of motion (North–South, East–West, and Vertical), ensuring a sufficiently broad and varied dataset for training.

As the initial class distribution was imbalanced (≈1:5 seismic to non-seismic), data augmentation techniques were implemented to enhance class uniformity. These techniques included variable-length spectrogram cropping and temporal shifts, resulting in physically consistent synthetic variations in the original signals. The final training dataset contained more than 60,000 spectrograms, each rescaled to a uniform dimension of 60 × 180 pixels.

**Figure 3:**
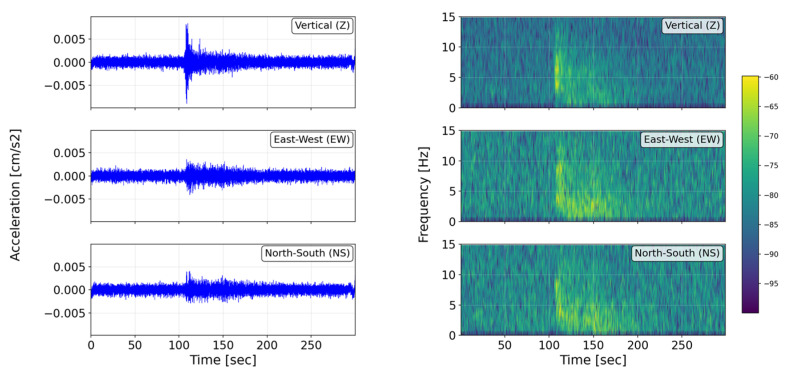
Acceleration time history and spectrogram of a seismic event record. The dark green color indicates the highest values.

The spectrograms were computed over the 0.1–25 Hz frequency band, which encompasses the dominant seismic energy range observed in the study region (i.e., the Peruvian territory). Within this band, time–frequency energy patterns clearly differentiate seismic events from ambient noise, as illustrated in Figure 3.

#### 2.3.2. Training Process

The training process was designed to enable the convolutional neural network (CNN) to automatically learn discriminative time–frequency patterns for the identification of seismic events. The classifier operates on spectrogram images computed independently for each of the three orthogonal components of motion (North–South, East–West, and Vertical).

Each spectrogram is normalized and resized to a fixed resolution of 60 × 180 pixels, corresponding to the frequency range 0.1–25 Hz and the temporal evolution of spectral energy. The resulting inputs are treated as single-channel images and processed individually by the CNN.

Table 1 summarizes the CNN architecture layer by layer and should be read from top to bottom. For each layer, the reported output dimensions correspond to the feature-map size obtained after applying convolutional and pooling operations to the input spectrogram of size 60 × 180 × 1. As detailed in Table 1, the resulting architecture comprises a total of 834,930 trainable parameters.

The CNN architecture is composed of three convolutional blocks. In each block, convolutional layers with ReLU activation extract relevant patterns from the spectrogram, while max-pooling operations reduce dimensionality by retaining the most informative features. Dropout regularization is applied after each pooling stage to improve robustness and mitigate overfitting. Through this hierarchical structure, the network progressively captures time–frequency features associated with seismic events. The output of the final convolutional block is flattened into a one-dimensional feature vector of 1600 elements, which is processed by a fully connected layer of 512 neurons. A softmax output layer with two neurons produces the probabilities corresponding to the seismic and non-seismic classes.

During training, the network parameters are optimized by minimizing a categorical cross-entropy loss function using the Adam optimizer. Gradients are computed through backpropagation and used to iteratively update the model weights.

**Figure 4:**
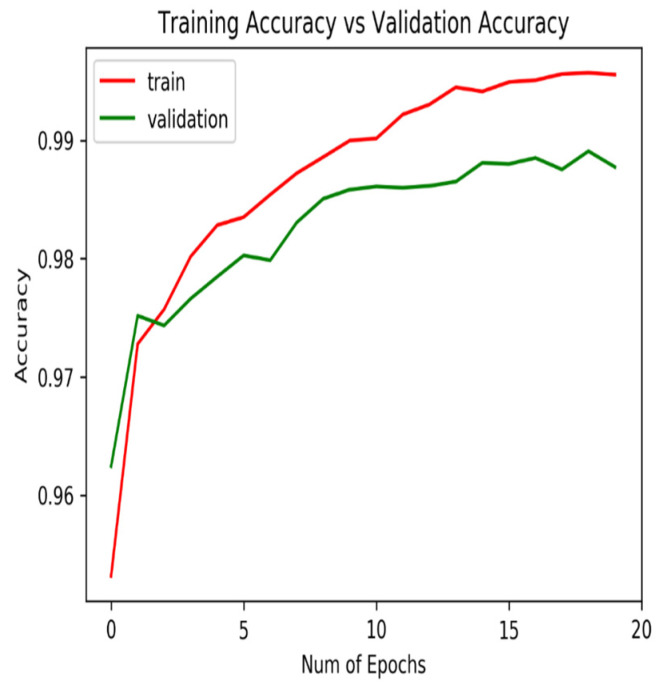
Training and validation accuracy evolution.

Model training was conducted using 70% of the available dataset, while the remaining 30% was reserved for validation. The trained model achieved a final training accuracy of 99.5% and a validation accuracy of 98.7%, indicating strong generalization capability with minimal overfitting, as illustrated in Figure 4.

#### 2.3.3. Performance of the Model

**Figure 5:**
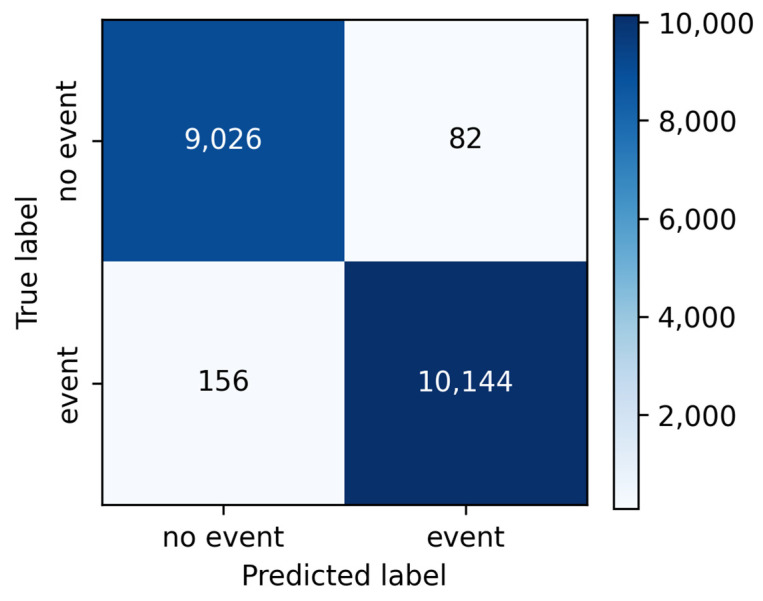
Confusion matrix of the classifier.

To further quantify the model’s performance, the precision, recall, and F1-score metrics were computed for each class, as presented in Table 2. The classifier achieved precision values exceeding 99% for the seismic event class, highlighting its reliability in distinguishing real earthquake signals from noise. The confusion matrix in Figure 5 reinforces the robustness of the model, showing few misclassifications.

Once validated, the CNN classifier was integrated into SIPA’s operational workflow. Each incoming accelerometric record is first evaluated by the classifier; signals identified as seismic proceed to the subsequent processing stages. The workflow begins with baseline correction to remove low-frequency drift.

A fourth-order Butterworth band-pass filter covering the 0.1–25 Hz range is then applied. This filter—selected for its smooth passband characteristics and absence of ripple—minimizes distortion while preserving the frequency content relevant for seismic analysis.

**Figure 6:**
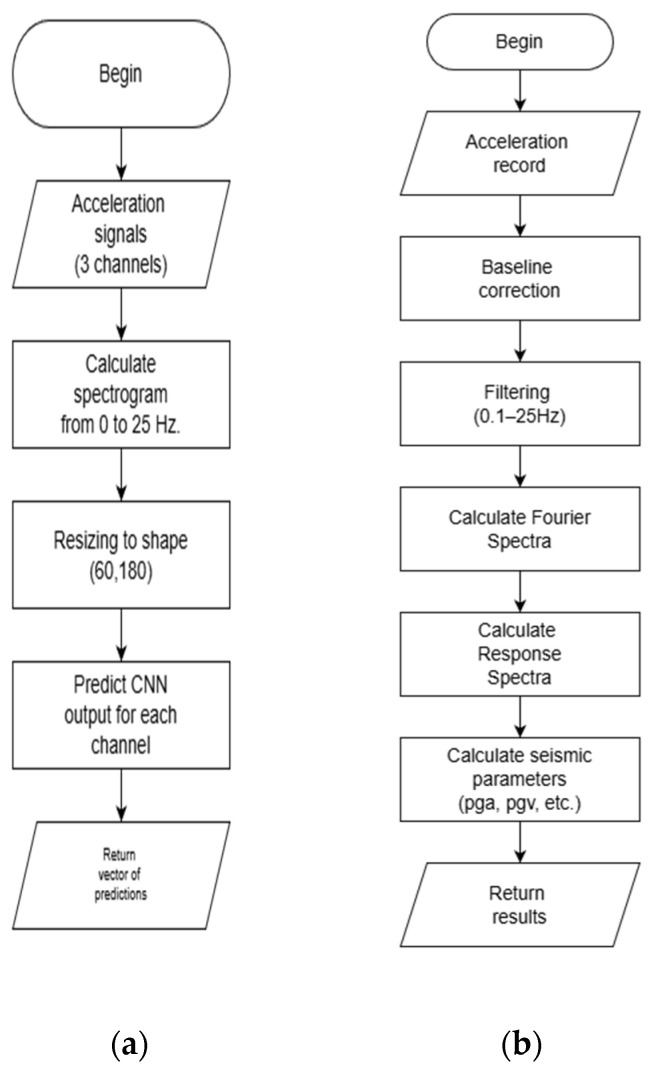
(**a**) Flowchart of the CNN-based classification algorithm; (**b**) Processing diagram from baseline correction and filtering to engineering parameters.

The classification algorithm flowchart is shown in Figure 6a, while Figure 6b details the processing sequence—from baseline correction and filtering to the computation of engineering parameters.

Within this workflow, SIPA processing follows a sequence of correction, filtering, and spectral analysis steps. First, the mean value is subtracted from each record to remove baseline offsets. Subsequently, a fourth-order Butterworth band-pass filter is applied in the 0.1–25 Hz frequency range. This filter preserves a smooth response within the frequency band of interest, avoiding distortion or ripple effects.

Using the filtered signals, the Fourier amplitude spectrum is computed through the Fast Fourier Transform (FFT). This spectrum allows the identification of dominant ground motion frequencies and serves as the basis for subsequent analyses.

The next step consists of obtaining response spectra by solving Duhamel’s integral for a second-order system using the eight-constant method.

Using the filtered signals, both the Fourier amplitude spectrum and the response spectrum are calculated, enabling the derivation of essential seismic engineering parameters: Peak ground acceleration (PGA), peak ground velocity (PGV), Arias intensity, maximum displacements, and specific energy density. The complete methodological sequence is illustrated in Figure 6b.

## 3. Results

### 3.1. Static Gravity Calibration

First, the static bias (offset) of each axis of the triaxial ADXL355 was verified using the gravity vector as a natural reference. This method does not require expensive external equipment and achieves good accuracy under controlled conditions.

The device was oriented sequentially in three positions:

- Z-axis vertical and upward (positive gravity);
- Z-axis vertical and downward (negative gravity);
- X and Y axes are oriented similarly by rotating the device about its axis.

These configurations allowed for the identification and correction of zero shifts (offsets) in each channel. The effective gain was also estimated by comparison against ±1 g.

Tests were conducted in a laboratory with stabilized temperature (±1 °C), minimizing thermal drift effects in the sensor. The controlled environment is crucial, as small temperature variations can affect the bias stability and sensitivity of the ADXL355.

The procedure was automated via a calibration script that records measurements at each position, then produces a correction parameter file. This file is loaded by the embedded system and applied in real time or during post-processing.

### 3.2. Dynamic Validation

**Figure 7:**
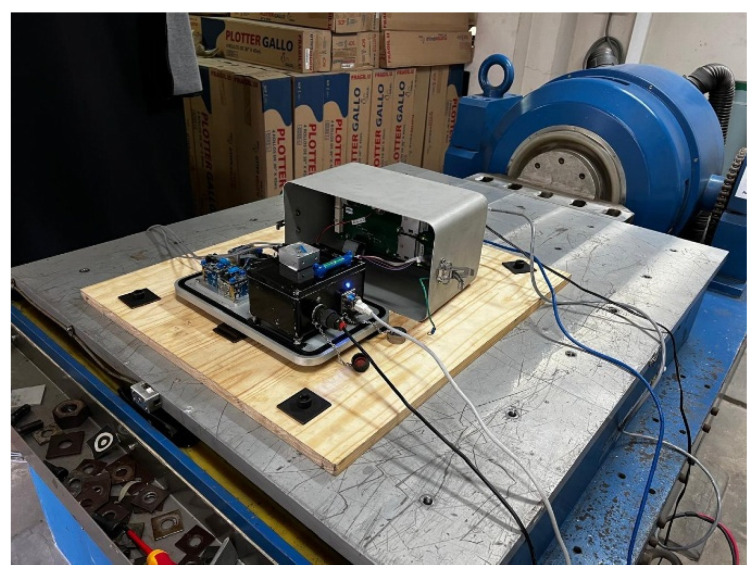
Shaking-table experimental setup at UNI with KUYUY and Kyowa AS-1TG mounted for identical excitation.

The dynamic validation was performed on the shaking table of one of the laboratories at the National University of Engineering (UNI), using the KUYUY accelerograph and the Kyowa AS-1TG accelerometric transducer as a reference sensor for the evaluation of the dynamic response (Figure 7). The Kyowa sensor was mounted directly on the upper surface of the KUYUY device, and both instruments were properly anchored to the shaking table to ensure identical input motion. The test campaign consisted of 12 controlled sinusoidal excitations at discrete frequencies spanning approximately 0.6 to 31.5 Hz (0.6, 0.9, 1.3, 1.8, 2.6, 3.7, 5.4, 7.6, 10.8, 15.4, 22.0, and 31.5 Hz). This controlled and repeatable configuration provides an adequate basis for evaluating the amplitude fidelity, spectral consistency, and phase behavior of the KUYUY accelerograph across the operational frequency range relevant for the recording of strong ground motions.

**Figure 8:**
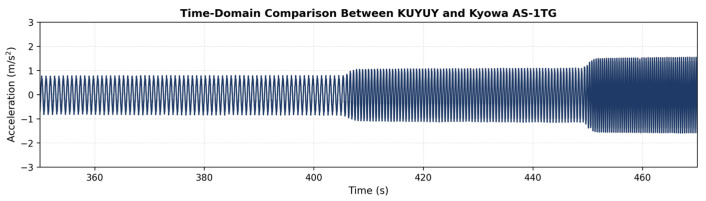
Time-domain acceleration comparison between KUYUY and Kyowa AS-1TG under sinusoidal excitations.

The recordings were synchronized using cross-correlation to ensure precise temporal alignment between the two sensors. The time-domain traces shown in Figure 8 indicate that, at low excitation frequencies, KUYUY and the Kyowa AS-1TG exhibit nearly identical response amplitudes and waveform shapes. In this frequency range, the peak-to-peak acceleration levels measured by both instruments are practically indistinguishable. This result demonstrates that KUYUY reproduces the input motion with amplitude fidelity comparable to the reference transducer. Such agreement at low frequencies confirms that KUYUY accurately captures the imposed motion under stable shaking-table operating conditions. This behavior provides a reliable foundation for the subsequent frequency response function (FRF) analyses.

**Figure 9:**
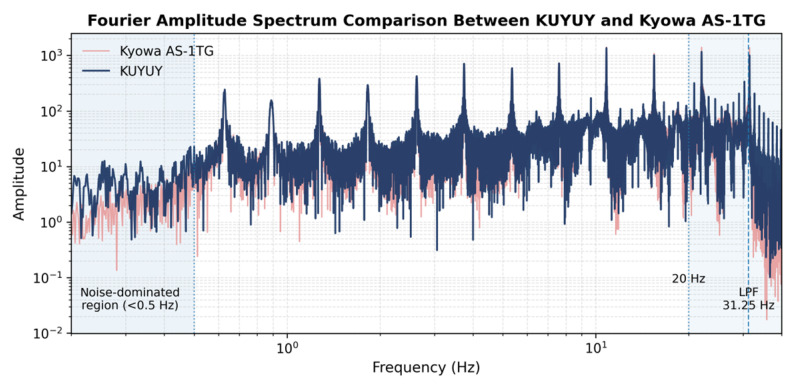
Fourier amplitude spectrum comparison between KUYUY and Kyowa AS-1TG under discrete sinusoidal excitation.

The Fourier amplitude spectra shown in Figure 9 largely confirm the agreement observed in the time domain; however, slight discrepancies appear at the lower and upper ends of the frequency range. At very low frequencies (below 0.5 Hz), the shaking table did not reach steady-state oscillation with sufficient amplitude, and the measured spectra are therefore dominated by sensor noise rather than true mechanical motion. Under these conditions, the apparent differences between KUYUY and the Kyowa AS-1TG reflect the comparison of their respective noise floors, rather than a mismatch in dynamic response.

At higher frequencies, particularly above approximately 12–15 Hz, KUYUY exhibits progressively more pronounced spectral peaks compared to the reference. This behavior becomes more evident beyond 20 Hz and is directly associated with the internal digital low-pass filter of the ADXL355 sensor, which has a cutoff frequency of 31.25 Hz. As the excitation frequencies approach this corner, the finite bandwidth of the digital signal chain introduces attenuation and spectral shaping effects that modify the amplitude response near the cutoff. These trends are consistent with the expected behavior of MEMS accelerometers operating close to the upper limit of their usable bandwidth and do not indicate instability or spurious signal generation.

**Figure 10:**
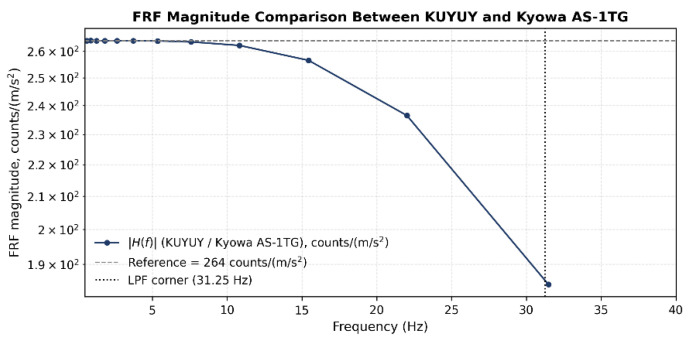
Frequency response function (FRF) magnitude comparison between KUYUY and the Kyowa AS-1TG, showing the reference sensitivity level and the internal low-pass filter corner at 31.25 Hz.

Amplitude sensitivity was quantified using the frequency response function (FRF), defined as the ratio between the acceleration amplitudes measured by KUYUY and the Kyowa AS-1TG at each excitation frequency. As shown in Figure 10, the FRF magnitude remains essentially constant at low frequencies, with values close to the nominal sensitivity ratio, indicating excellent amplitude agreement between both instruments. As frequency increases, the FRF exhibits a smooth and progressive reduction, which becomes more evident beyond approximately 15–20 Hz. This behavior is primarily associated with the finite bandwidth of the sensing system and the internal digital low-pass filter of the ADXL355, whose corner frequency is 31.25 Hz. The absence of abrupt deviations or irregular oscillations confirms that the observed attenuation follows a predictable and physically consistent trend, supporting the validity of KUYUY’s dynamic response within the frequency range relevant for the recording of strong ground motions.

**Figure 11:**
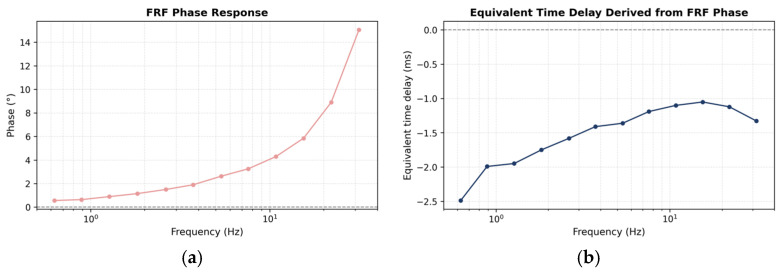
Frequency response function (FRF) phase response and equivalent time delay between KUYUY and the Kyowa AS-1TG: (**a**) FRF phase as a function of frequency and (**b**) equivalent time delay derived from the phase response.

The phase component of the frequency response function provides additional insight into the dynamic behavior of the KUYUY accelerograph relative to the Kyowa AS-1TG. As shown in Figure 11a, the FRF phase exhibits a smooth and monotonic increase with frequency, indicating a progressively larger phase lag of KUYUY with respect to the reference sensor. This behavior is characteristic of low-mass MEMS accelerometers, where the finite compliance of the proof-mass suspension and the internal signal-conditioning stages introduce frequency-dependent phase delays. No abrupt phase jumps or oscillatory artifacts are observed, confirming the stability and linearity of the system response.

To aid physical interpretation, the phase response was converted into an equivalent time delay, shown in Figure 11b. The resulting delay remains small and varies smoothly across the analyzed frequency range, with values on the order of a few milliseconds. Such delays are negligible, do not compromise waveform coherency, and do not affect the recording of strong ground motions, as they fall outside the range of dominant periods associated with this type of signal. The consistency of both the phase and equivalent delay trends confirms that KUYUY behaves as a stable, lightly damped electromechanical system, suitable for accurate dynamic measurements within the frequency band of interest.

The results obtained from the controlled shaking-table experiments demonstrate that the KUYUY accelerograph exhibits a dynamic response closely aligned with that of the Kyowa AS-1TG across the evaluated frequency range. Agreement in the time domain, consistency in the Fourier amplitude spectra, and the stable behavior observed in the FRF magnitude, phase, and equivalent time delay collectively confirm the accuracy and reliability of KUYUY under dynamic excitation. The observed amplitude attenuation and phase lag at higher frequencies follow predictable trends associated with the finite bandwidth and internal signal conditioning of MEMS sensors. Overall, these findings validate the suitability of KUYUY for the recording of strong ground motions within the frequency range of engineering interest.

### 3.3. Validation Against a Reference Accelerograph

**Figure 12:**
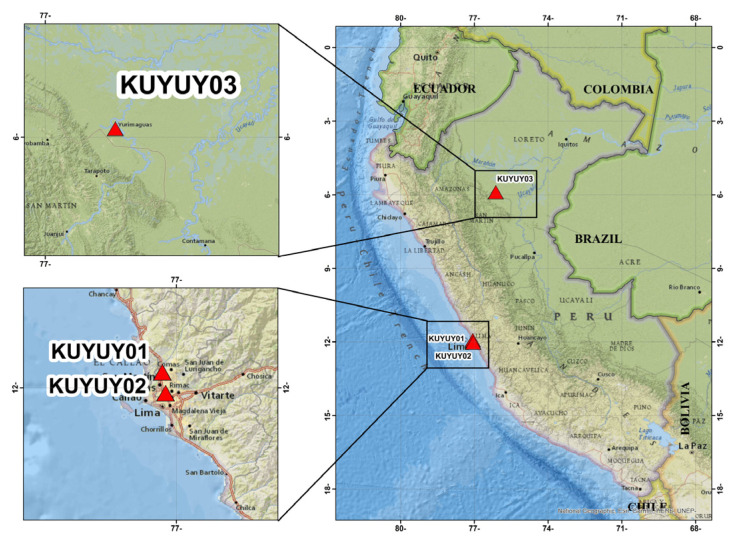
Locations of KUYUY accelerograph stations currently installed in Peru. KUYUY01 and KUYUY02 are in Metropolitan Lima, and KUYUY03 is in Yurimaguas.

After static calibration, the device underwent dynamic validation via comparison with a high-end commercial accelerograph. The KUYUY system was installed alongside a REFTEK 130-SMHR—a well-recognized instrument for seismic applications. The locations of the KUYUY01, KUYUY02, and KUYUY03 stations are shown in Figure 12, highlighting their deployment in Metropolitan Lima and Yurimaguas.

### 3.4. Field Testing in Real Scenarios

#### Field Records and Detected Events

At the time of the study, two KUYUY units were operational in Metropolitan Lima: KUYUY01 was installed in the Comas district, and KUYUY02 in the Rímac district. Between 15 and 17 June 2025, SIPA detected the events summarized in Table 3.

### 3.5. Results from the Intelligent Accelerographic Processing System (SIPA)

SIPA produced and published 1745 accelerographic reports on the project website for 2017–2019. Table 4 and Table 5 present report statistics for magnitude range, including ML (local magnitude), calculated from the maximum amplitude recorded by nearby seismographs, and Mw (moment magnitude), derived from the seismic moment that describes the energy released by fault rupture. The tables also organize the data according to the recorded peak acceleration values.

**Figure 13:**
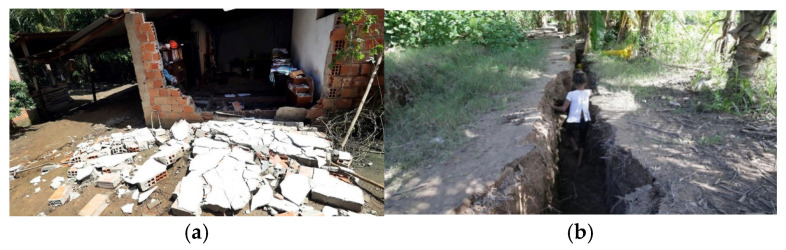
Damage observed after the Lagunas earthquake (26 May 2019): (**a**) Ground failure in the Seis de Enero population center; (**b**) Collapsed dwelling in Sauce.

As a representative case, we present the report for the 26 May 2019 Lagunas earthquake (02:41 local time) in northeastern Peru (Lagunas, Alto Amazonas, Loreto) with magnitude Mw 8.0. The event was associated with an intermediate-depth intraplate normal fault at approximately 110 km [6]. The reported impacts included 4562 people affected, 131 houses destroyed, 1270 houses rendered uninhabitable, and 40 rural roads destroyed [7]; examples of damage due to this earthquake are shown in Figure 13.

**Figure 14:**
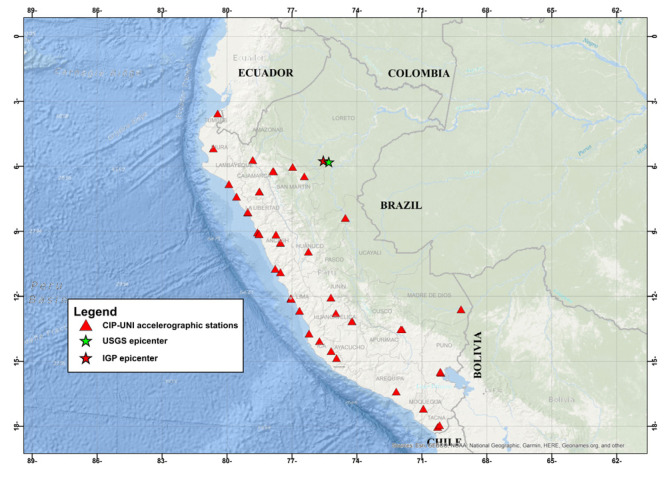
Stations that recorded the Lagunas earthquake on 26 May 2019.

SIPA recorded the event at 44 stations (Figure 14), with a maximum acceleration of 95.84 cm/s^2^. The four stations with the highest recordings are listed in Table 6, which also includes Arias intensity ($I_{\alpha }$) and specific energy density (SED); for comparison, the record from the CIP Lima station on stiff soil is included.

**Figure 15:**
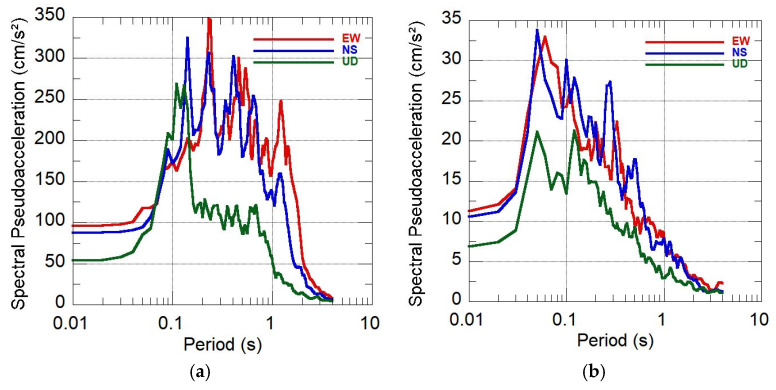
Response spectra for the Lagunas earthquake (26 May 2019) under contrasting soil conditions: (**a**) UNTRM station (soft soil); (**b**) CIP Lima station (stiff soil).

SIPA also generated a report [10] detailing accelerations, velocities, displacements, Fourier spectra, and response spectra for each station, using records obtained across multiple Peruvian departments, which enabled a comparison of dynamic behaviors across soil types. As an example, Figure 15 shows the response spectra for a soft clayey soil (UNTRM station, $d_{\mathrm{epi}}=292$ km, PGA=95.84 cm/s^2^) and a stiff gravelly soil (CIP Lima station, $d_{\mathrm{epi}}=733$ km, PGA=11.06 cm/s^2^). Despite the difference in epicentral distance, the spectra allow observation of the relative dynamic response of soft versus stiff soils under both high and low excitation levels.

## 4. Discussion of Results

During testing, we evaluated the device’s capability for continuous operation over more than eight hours without intervention, including validation of the integrity of the transmitted data and FTP communication stability. The recorded signals showed coherence among orthogonal components, with no evidence of orientation errors.

**Figure 16:**
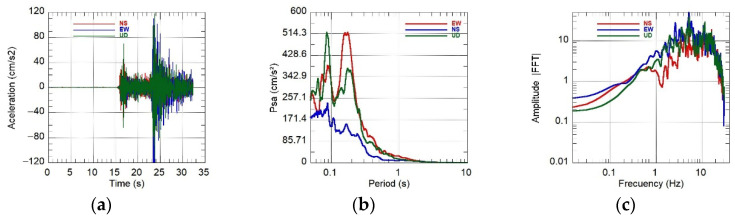
Event 15 June 2025 16:35:30 (Mw 5.6). Left to right: accelerations (3 axes), pseudo-acceleration spectrum PSa (log_10_ scale in T), and Fourier amplitude spectrum (log–log). Panels: (**a**) acceleration vs. time; (**b**) PSa (5% damping ξ); (**c**) Fourier amplitude spectrum.

**Figure 17:**
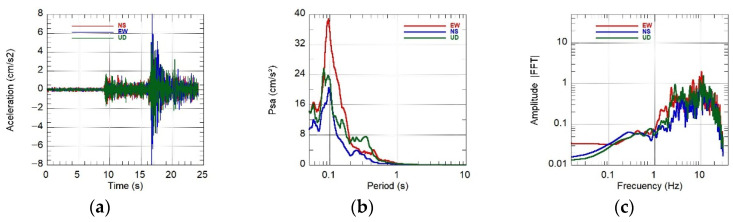
Event 16 June 2025 17:40:17 (ML 4.2). Left to right: accelerations (3 axes), pseudo-acceleration spectrum PSa (log_10_ scale in T), and Fourier amplitude spectrum (log–log). Panels: (**a**) acceleration vs. time; (**b**) PSa (5% damping ξ); (**c**) Fourier amplitude spectrum.

**Figure 18:**
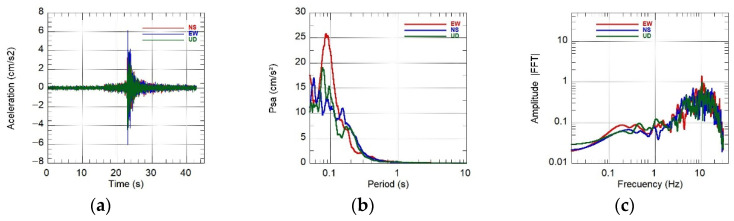
Event 17 June 2025 05:31:31 (ML 3.7). Left to right: accelerations (3 axes), pseudo-acceleration spectrum PSa (log_10_ scale in T), and Fourier amplitude spectrum (log–log). Panels: (**a**) acceleration vs. time; (**b**) PSa (5% damping ξ); (**c**) Fourier amplitude spectrum.

For the Mw 5.6 event on 2025-06-15, the KUYUY stations recorded PGA values of 156.9 cm/s^2^ (KUYUY01) and 123.2 cm/s^2^ (KUYUY02). Co-location of KUYUY01 with a RefTek SMHR allowed for quantitative verification of amplitude consistency. To minimize orientation and mounting stiffness effects, the comparisons used scalar metrics (${PGA}_{max}$ and ${PGA}_{V}$). The results are reported in Table 7 and Figure 16a–c, Figure 17a–c and Figure 18a–c.

Field tests and co-location indicated that KUYUY accurately reproduces horizontal amplitudes, with differences of only 1–2% for the Mw 5.6 event. For the ML 4.2 and ML 3.7 events, the discrepancies were 5–10%. To reduce biases due to minor orientation differences or mounting rigidity, we report scalar metrics: (i) ${\mathrm{PGA}}_{max}=max\left({\mathrm{PGA}}_{E-O},{\mathrm{PGA}}_{N-S}\right)$ and (ii) ${\mathrm{PGA}}_{V}$. The relative bias is defined as 100×(KUYUY01−RefTek)/RefTek.

The observed discrepancies in ${PGA}_{V}$ were consistent with typical vertical coupling differences in co-located surface installations. Overall, the results confirm that the proposed instrument is characterized by temporal stability, robustness of acquisition and transmission, and amplitude fidelity comparable to that of a recognized commercial reference instrument, supporting its suitability for integration into urban monitoring networks.

Regarding SIPA, the 26 May 2019 (Mw 8.0) Lagunas earthquake was key for system validation. The event was recorded at 44 stations (see Table 6 in Results). UNTRM registered PGA = 95.8 cm/s^2^, while values in Lima on stiff soils did not exceed 11 cm/s^2^. Energy-related parameters—including Arias intensity and specific energy density—were highest in cities such as Moyobamba and Tarapoto (see Table 6). These values correlate with the reported damage to housing and rural infrastructure in Loreto and San Martín (Figure 13). The correlation between instrumental records and observed effects underscores SIPA’s utility for emergency management and prioritization of post-earthquake interventions.

Moreover, reports were generated and published in under thirty minutes after the event, representing a substantial gain over traditional manual workflows. The combination of robust acquisition, stable transmission, and intelligent processing gives the system a decisive advantage regarding the provision of timely, reliable information, consolidating SIPA’s role in seismic risk reduction in Peru and demonstrating its potential for expansion to denser urban networks.

## 5. Conclusions

The static gravity calibration allowed accurate estimation of per-axis offsets and effective gains of the ADXL355 sensor. The controlled shaking-table experiments conducted at the National University of Engineering (UNI) confirm that the KUYUY accelerograph provides a dynamic response that is closely aligned with that of the reference Kyowa AS-1TG accelerometric transducer within the frequency range relevant to strong ground motions.

Field tests conducted in Lima further confirmed KUYUY’s operational robustness under real conditions. The instruments maintained continuous operation, stable telemetry, and data transmission integrity, successfully recording four representative earthquakes that occurred in June 2025. Additionally, the performance of the SIPA system had already been validated during a major real event: the accelerographic report generated for the 26 May 2019 Lagunas earthquake directly supported the institutions responsible for Seismic Risk Management during the immediate post-event response, demonstrating the early maturity of the automatic pipeline and its operational usefulness in emergency contexts. Furthermore, SIPA’s detection module, based on deep neural networks for discriminating seismic and non-seismic signals, achieved an accuracy close to 98%, confirming the effectiveness of this approach and its potential for improvement as more labeled data become available.

These results are particularly relevant in the Peruvian context, where significant gaps persist in the spatial density of accelerographs and in the instrumentation of buildings. The earthquakes of 2001, 2007, and 2019 highlighted the need for dense networks and timely telemetry. Although force-balance accelerographs remain the international standard due to their wide dynamic range and long-period stability, their high acquisition and maintenance costs limit large-scale deployment in the country. Under these constraints, the KUYUY accelerograph—low-cost, low-noise, and equipped with real-time telemetry—integrated with the SIPA automatic processing system, represents a viable and technically consistent solution for increasing the national seismic monitoring capacity. Its interoperability with automatic analysis pipelines facilitates microzonation studies and rapid damage assessments, contributing directly to seismic risk reduction.

Overall, the evidence presented demonstrates that KUYUY, in combination with the SIPA system, constitutes a reliable, accurate, and operationally robust platform for seismic monitoring. International experience with dense arrays and current advances in instrumentation technologies support this integrated proposal, which provides a practical, scalable, and sustainable pathway toward strengthening seismic monitoring in Peru and moving toward future dense, real-time urban networks.

## 6. Patents

This work includes a utility model patent for the Kuyuy device, filed in 2025. The equipment described in this manuscript is currently in the final stages of the registration process.

This work includes a utility model patent application for the KUYUY device, filed in July 2025 in Peru. The application is in the final stages of the registration process.

## Figures and Tables

**Table 1:** CNN architecture used for seismic signal classification.

Layer	Output	Parameters
Conv2D_1	(58, 178, 32)	320
MaxPooling2D_1	(29, 89, 32)	0
Dropout_1	(29, 89, 32)	0
Conv2D_2	(27, 87, 32)	9248
MaxPooling2D_2	(13, 43, 32)	0
Dropout_2	(13, 43, 32)	0
Conv2D_3	(11, 41, 16)	4624
MaxPooling2D_3	(5, 20, 16)	0
Dropout_3	(5, 20, 16)	0
Flatten	(1600)	0
Dense_1	(512)	819,712
Dense_2	(2)	1026
Total	—	834,930

**Table 2:** Classification metrics for the validation set.

Class	Precision	Recall	F1-Score
No Event	0.9830	0.9910	0.9870
Event	0.9920	0.9849	0.9884

**Table 3:** Events detected by KUYUY stations (maximum values per station).

Device	Date (UTC)	Magnitude	$d_{\mathrm{epi}}$ (km)	PGA (cm/s^2^)
KUYUY01	15 June 2025 16:35:30, Callao	5.6 Mw	45.5	156.9
KUYUY01	16 June 2025 17:40:17, Callao	4.2 ML	46.6	9.2
KUYUY01	17 June 2025 05:31:31, Ancón	3.7 ML	41.6	6.1
KUYUY02	15 June 2025 16:35:30, Callao	5.6 Mw	41.0	123.2

**Table 4:** Number of accelerographic reports by magnitude.

Magnitude Range	Reports (n)	Accelerographs (n)		
		min	mean	max
ML≤5	1619	1	3.05	26
5<Mw≤7	123	1	8.21	34
Mw>7	3	28	34.66	44

**Table 5:** Number of accelerographic reports by peak acceleration.

Acceleration Range (cm/s^2^)	Reports (n)
[0,10]	1595
(10,50]	135
(50,100]	10
(100,400]	5

**Table 6:** Stations with the highest recordings during the 2019 Lagunas event and reference record at CIP Lima.

Station	Comp.	PGA (cm/s^2^)	PGV (cm/s)	PGD (cm)	$V_{max}/A_{max}$ (s)	RMS Acc (g)	RMS Vel (cm/s)	RMS Disp (cm)	$I_{\alpha }$ (m/s)	SED (m^2^/s)
UNTRM	EO	95.84	13.46	2.62	0.14	11.06	1.32	0.29	0.87	772.04
	NS	87.45	8.34	1.49	0.10	10.64	1.06	0.20	0.81	501.81
	V	53.49	3.52	0.92	0.07	6.64	0.44	0.12	0.31	84.23
CIP MOYOBAMBA	EO	91.29	19.13	6.62	0.21	12.96	3.40	1.51	0.81	3469.17
	NS	78.76	20.85	9.14	0.26	12.39	3.48	1.64	0.74	3623.72
	V	90.16	13.08	4.21	0.15	11.43	2.09	0.70	0.63	1312.25
CIP TARAPOTO	EO	58.18	9.00	3.04	0.15	14.15	2.03	1.07	0.35	451.00
	NS	79.56	10.70	3.34	0.13	16.19	2.13	0.79	0.46	491.97
	V	67.86	4.74	1.26	0.07	10.56	1.03	0.38	0.19	115.30
CIP AMAZONAS	EO	78.91	7.32	1.70	0.09	7.79	0.79	0.20	0.43	275.13
	NS	53.98	5.84	1.11	0.11	7.16	0.75	0.17	0.36	249.44
	V	53.05	3.07	0.93	0.06	5.26	0.41	0.13	0.20	75.30
CIP LIMA	EO	11.06	0.74	0.28	0.07	0.92	0.08	0.06	0.01	2.89
	NS	10.43	0.54	0.26	0.05	0.88	0.07	0.04	0.01	2.11
	V	6.78	0.37	0.17	0.05	0.68	0.05	0.04	0.00	1.25

**Table 7:** KUYUY01 vs. RefTek SMHR for co-located events. Relative bias is 100×(KUYUY01−RefTek)/RefTek.

Event (UTC)	Mag.	PGA_max_ (cm/s^2^)			PGA_V_ (cm/s^2^)		
		KUYUY01	RefTek	Deviation (%)	KUYUY01	RefTek	Deviation (%)
15 June 2025 16:35:30	Mw 5.6	156.9	159.0	−1.3	117.5	114.5	+2.6
16 June 2025 17:40:17	ML 4.2	9.2	9.8	−6.1	5.3	4.5	+17.8
17 June 2025 05:31:31	ML 3.7	6.1	6.4	−4.7	4.5	4.2	+7.1

## Data Availability

The data presented in this study are available from the corresponding author upon reasonable request due to privacy considerations.
